# Editorial: Genomics in plant sciences: understanding and development of stress-tolerant plants

**DOI:** 10.3389/fpls.2023.1222818

**Published:** 2023-06-07

**Authors:** Ayaz Mahmood Dar, Hussain Touseef, Kashif Nawaz, Yusuf Khan, Pranav Pankaj Sahu

**Affiliations:** ^1^ Organic and Biomolecular Research, Department of Higher Education (GDC Sogam), University of Kashmir, Srinagar, Jammu and Kashmir, India; ^2^ Sevama AgriClinic and Laboratory, MatI Mate Agromart Pvt. Ltd., Bhavnagar, Gujarat, India; ^3^ Department of Plant Breeding and Genetics, Max Planck Institute for Plant Breeding Research, Köln, Germany; ^4^ Biological and Environmental Science and Engineering Division, King Abdullah University of Science and Technology, Thuwal, Saudi Arabia; ^5^ Newborn Screening Department, University Hospital, Oslo, Norway; ^6^ Laboratory of Ecological Plant Physiology, Global Change Research Institute of the Czech Academy of Sciences, Brno, Czechia

**Keywords:** genomics, crop genetics, breeding, genome-wide studies, multi-omics, abiotic, biotic, stress-tolerance

Environmental stresses have a profound impact on the growth and productivity of plants (Boyer, 1982). The predicted growth of the human population and impending changes in climate could further threaten food availability around the World. Hence, it is of prime importance for researchers across the world to fast-track the development the high-yield stress-tolerant plants. The process requires a deeper understanding of genome structure, and biochemical, physiological, and molecular mechanisms underlying abiotic and biotic stress tolerance in plants. Plants have evolved complex molecular mechanisms to respond to both these stimuli (Bechtold and Field, 2018). It comprises the recognition of stress factors, activation of the stress signaling cascade, and synthesis of stress-related genes, proteins, or other molecules (Zhang et al., 2020). Moreover, genomics approaches like genetic diversity analysis, allelic variations identification, phylogenies, and understanding the adaptive evolution of genomes could also serve as vital tools to develop plant varieties mitigating food security issues (Cushman and Bohnert, 2000). Hence, an extensive effort is needed to utilize the plant genomics resources generated and to identify the key genes and regulators, which can withstand the diverse agroecological conditions/climates. This Research Topic represented the collection of nine original research articles and two reviews signifying the role of genomics, functional genomics, and multi-omics approaches in the development of stress-tolerant plants.

The identification of hotspots in the genomic architecture of plant species by chromosome-scale genome assembly, comparative genomics, and multi-omics approach could assist in mapping stress-associated genes and quantitative trait loci (QTLs). The captured QTLs and SNPs markers in the genome not only provide information about the genomic architecture but could impart stress resilience in plants by marker-assisted selection and breeding. A study done by Sarkar et al. on maize successfully mapped the QTLs for the growth- and yield-related traits under water availability scenarios. The genes underlying these QTLs have specific functions in maintaining the growth and senescence, and hormone signaling under water deficit conditions. The nucleotide polymorphism in the promoter elements in rice has been shown to modulate the salt-responsive attributes by Haque et al. The comparative sequence analysis of K^+^ salt-responsive transporters (*OsTPKa* and *OsHAK*) among two contrasting rice genotypes differed in salt-responsiveness revealing that both have unique TF-binding motifs. These modifications in the elements, played a key role in the coordinated expression of both transports, leading to an effective acclimation response in rice.

The candidate genes and proteins belonging to the flavonoid, phenylpropanoid, and α-linolenic acid metabolism were mapped on the tea (*Camellia sinensis*) genome by Wang et al. These potential targets in the tea genome could play an imperative role in the development of tea plants resistant to green leafhopper infestation. A similar approach was also applied to understand the genetic basis of cold adaptation in Asparagus beans by Liang et al. In the comparative genomics approach between contrasting bean accessions, a set of variable genes in two individual genomes was retrieved. A detailed analysis of these genes identified that in the genome of cold tolerant accession, Ningjiang 3 the presence of ABCC-type transports (a sub-gene family of ABC transporters) was linked with enhanced abiotic stress resilience.

A massive structural variation in the sesame pan-genome due to evolutionary adaptations was reported by Parakkunnel et al. These genetic novelties in APETALA2/Ethylene Responsive Factor (AP2/ERF) and WRKY transcription factors were due to alterations in the copy numbers, shifts in their location, and structural changes in the *cis*-element and exon-intron structure. These transcription factors (TFs) have a very specific role in the regulation of plants’ defense against environmental stress, hence suggesting the adaptive selection pressure leading to structural variation in the sesame genome. Similarly, the comparative analysis of the chloroplast genome of black pepper (*Piper nigrum L*.) with related piper species was studied by Gaikwad et al. The study provided key insights into the chloroplast genome evolution, which will assist in the development of molecular markers and accelerate ongoing black pepper improvement programs.

Moreover, *in-silico* genome-wide analysis of key gene families and their comparative analysis could decipher the biotic and abiotic stress adaptations in plant species. A similar strategy was further exploited by Nie et al., to explore the role of leucine-rich repeat receptor-like kinases (LRR-RLKs) during *Bursaphelenchus xylophilus* infestation in *Pinus massoniana*. The candidate RLK genes retrieved from the transcriptome datasets were subjected to structural and molecular characterization. The study revealed that an LRR XII subfamily gene *PmRLKs32*, having ROS-producing properties played an important role in providing nematode resistance in pine trees. An extensive structural, functional, molecular, and evolutionary study on the Caffeoyl-CoA O-methyltransferase (CCoAOMT) gene family was carried out in two jute species by Akhter et al. Despite both species showing high syntenic conservation among *CCoAOMTs*, the gene expression level was modulated among them under various abiotic stresses and hormonal treatment. Hence, the key gene from the CCoAOMT gene family could be used to genetically engineered jute plants that can withstand a wide range of environmental stresses. Though, it is also important to functionally characterize the candidate genes for the required traits. For example, Zhang et al., showed how an MYC TF plays a major role in regulating growth and fruit quality in tomato. Through molecular study, they reported that the interplay between MYC2 and jasmonic acid (JA) and TOR signaling pathways regulates the growth and development of tomato seedlings.

This Research Topic contains two important review articles focused on genetic (structural changes) and epigenetic (reversible changes) modifications in the genome leading to stress adaptations in plants. The retrotransposons are varied in copy number and activated by various biotic and abiotic stresses due to retrotransposition bursts. The review by Papolu et al., focused on the dynamics of LTR retrotransposons and the associated mechanism of genome expression and adaptive evolution during high-temperature stress. The epigenetic changes are transgenerational and important for the stress memories in plants (Sahu et al., 2013). The review by Ramakrishnan et al., summarized the regulatory mechanism of memory establishment in plants under cold and heat stress, through histone modification and DNA methylation process.

Overall, the present research studies highlight the importance of genetic and genomic tools in the identification and development of stress-tolerant plants. The progress made towards understanding how structural changes in plant genomes could shape the unique response to environmental stresses will be critical for the improvement of crop plants.

## Author contributions

All authors have contributed in writing, editing, reading and agreed to the final version of the manuscript.
